# Validation of the AViTA BPM636 upper arm blood pressure monitor in adults and pregnant women according to the ANSI/AAMI/ISO 81060-2:2013

**DOI:** 10.1097/MBP.0000000000000648

**Published:** 2023-04-05

**Authors:** Chien-Nan Lee, Cho-Kai Wu, I-Chih Huang

**Affiliations:** a Department of Obstetrics & Gynecology, National Taiwan University Hospital; b Division of Cardiology, National Taiwan University Hospital; c R&D Software Department, AViTA Corporation, Taipei City, Taiwan

**Keywords:** ANSI/AAMI/ISO 81060-2, AViTA, blood pressure monitor

## Abstract

To evaluate the accuracy of the AViTA oscillometric upper arm home blood pressure (BP) monitor in adult and pregnant populations according to the American National Standards Institute/Association for the Advancement of Medical Instrumentation/ International Organization for Standardization (ANSI/AAMI/ISO) Universal Standard (ISO 81060-2:2013).

**Methods:**

BP measurements on the upper arm were performed on 85 adult subjects and 46 pregnant subjects. The AViTA BPM636 and a standard mercury reference sphygmomanometer were applied and followed the same arm sequential BP measurement method. The universal cuff of the test device was used for arm circumference of 22–42 cm.

**Results:**

For validation criterion 1, the mean ± SD of the differences between the test device and reference BP readings was 1.1 ± 5.49/2.9 ± 5.17 mmHg (systolic/diastolic) for adults; and -2.2 ± 5.93/1.5 ± 4.92 mmHg (systolic/diastolic) for pregnant women. For criterion 2, the SD of the averaged BP differences between the test device and reference BP per adult subject was 4.45/4.20 mmHg (systolic/diastolic) and per pregnant women was 4.66/3.96.

**Conclusion:**

The AViTA BPM636 had passed the criteria of the ANSI/AAMI/ISO 81060-2:2013 protocol and can be recommended for home BP measurements in adults and pregnant populations.

## Introduction

Hypertension is one of the top causes of death and disease [1]. If hypertension can be easily detected by measuring blood pressure (BP) at home, it may often be treated effectively with low-cost medications [2]. Gestational hypertension and pre-eclampsia mainly cause maternal and perinatal morbidity and mortality worldwide [3]. Early detection of gestational hypertension and BP management before delivery are important for pregnant women [4].

The mercury sphygmomanometer, which is one of the earliest devices used to measure BP, can be traced back to the late 19th. After 1978s, automated electronic BP monitors have been used in clinical practice, particularly in home self-measurement of BP. And now, home BP measurement has been used in clinical practice [5]. Various guidelines for managing hypertension recommend using automated BP measuring devices [6,7]. The condition is that these devices must be properly validated for accuracy according to standardized protocols [8–15]. However, few devices have been validated, and rare have passed the validation without violating the guideline. It reflects the fact that many automated BP monitors failed to pass the proper validation, and also emphasizes the importance of such a validation process.

In this study, the arm type BP device (AViTA Co., Ltd., Taipei, Taiwan) was applied and measured BP by an oscillometric method in adult subjects and pregnant women. It was evaluated according to the criteria of ANSI/AAMI/ISO 81060-2:2013.

## Material and methods

### Test device

The BPM636 (AViTA Co., Ltd.) is an oscillometric upper arm BP monitor intended for self- BP measurement at home. The device has a semi-conductive pressure sensor designed to measure static pressure values ranging from 0 to 300 mmHg and pulse rate values from 40 to 199 beats per minute. The declared specific accuracy is ±3 mmHg for pressure and ±4% for pulse rate. The device has two memory zones and each comprises a memory capacity of 60 measurements. The device requires four 1.5 V AA (LR6) alkaline batteries as an energy source. At least 300 BP readings can be obtained with each battery set. The universal adult-cuffs (Easy Cuff) are suitable for arm circumferences from 22 to 42 cm. During the measurement period, the forearm and the device should be on a table with the palm facing up and the device at the heart level. The manufacturer provided test monitors in three units which were randomly selected for the validation process. The method follows the section 5.2.4.2 of ANSI/AAMI/ISO 81060-2 noninvasive sphygmomanometers validation protocol [16–18]. The same arm sequential method was implemented with AViTA Easy Cuff Size of 22–42 cm to measure systolic and diastolic BPs.

### Reference device

Reference measurements were performed by a mercury sphygmomanometer (KENLU-K300, KENLU, Kaohsiung City, Taiwan). The evaluator-blinded procedure was designed through two trained and experienced observers who were blinded from each other’s reading. A double stethoscope was used to exclude the poor Korotkoff sounds. A third observer recorded the pair BP values of two observers.

### Recruitment

The study was conducted from 23 October 2018, to 25 September 2019. Among 132 participants were recruited from the staff and hypertensive patients in National Taiwan University Hospital (NTUH), one participant was excluded due to arrhythmia found during the measurement process. A total of 131 participants were qualified for the inclusion criteria: (1) group A: 45 male (53%) and 40 female (47%) adult subjects were recruited, and (2) group B: 46 pregnant women were enrolled, including 15 normotensive pregnant subjects with negative proteinuria test; 16 hypertensive pregnant patients with elevated SBP ≥ 140 mmHg or DBP ≥ 90 mmHg and negative/trace proteinuria test results; and 15 pre-eclampsia patients with elevated DBP ≥ 90 mmHg and proteinuria >300 mg in 24 h. Individuals with cardiac arrhythmias, patients with known heart disease, or diagnosed with secondary hypertension were excluded from group A and group B. The clinical characteristics of the participants and the distribution of reference BP measurements in the study are summarized in Tables 1 and 2. The characteristics results were show with the requirements of validation protocol. The cuff size of participate in adults and pregnancy women groups were measured and displayed in Table 3.

**Table 1:** Characteristics of the study participants

Participants’ characteristics	Group 1 (*N* = 85)		Group 2 (*N* = 46)	
	Mean ± SD	Range	Mean ± SD	Range
Age (years old)	51.6 ± 18.5	22–89	35.6 ± 4.7	27–45
Arm circumference (cm)	30.9 ± 4.9	23–39.5	28.1 ± 4.1	23–39.5
Entry SBP R0 (mmHg)^a^	126.8 ± 22.4	86–192	137.6 ± 25.1	86–185
Entry DBP R0 (mmHg)^a^	79.1 ± 13.8	48–119	92.3 ± 17.5	58–121
Gender				
Male	45 (53%)		-	
Female	40 (47%)		46 (100%)	
Normotensive	59 (69%)		15 (33%)	
Hypertensive^b^	26 (30%)		16 (34%)	
Pre-eclampsia^c^	-		15 (33%)	

a Mean ± SD.

b Group 1: SBP≥140 mmHg or DBP≥90 mmHg. Group 2: (i) SBP≥140 mmHg or DBP≥90 mmHg, and (ii) without proteinuria >300 mg in 24 hr.

c Group 2: (i) DBP≥90 mmHg, and (ii) with proteinuria >300 mg in 24 hr.

**Table 2:** Distribution of reference blood pressure measurements

Blood pressure distribution		Group 1 (*n* = 255)^b^	Group 2 (*n* = 138)^c^
SBP^a^	≤100 mmHg	28 (11%)	20 (14%)
	≥160 mmHg	23 (9%)	24 (17%)
	≥140 mmHg	64 (25%)	70 (51%)
DBP^a^	≤60 mmHg	20 (8%)	7 (5%)
	≥100 mmHg	20 (8%)	52 (38%)
	≥85 mmHg	89 (35%)	102 (74%)

a Number of recordings.

b A total of 255 valid paired blood pressure values from 85 subjects in group 1.

c A total of 138 valid paired blood pressure values from 46 subjects in group 2.

**Table 3:** Blood pressure cuff size distribution of patients

Group 1	Arm circumference	Number of subjects
Adult participants (*N* = 85)		
Single cuff (22–42 cm)	Small (22–27 cm)	21 (24.7%)
	Adult (27–32 cm)	22 (25.88%)
	Large Adult (32–42 cm)	42 (49.41%)

Group 2	Arm circumference	Number of subjects
Pregnant participants (*N =* 46)		
Single cuff (22–42 cm)	Small (22–27 cm)	22 (47.82%)
	Adult (27–32 cm)	18 (39.13%)
	Large adult (32–42 cm)	6 (13.04%)

### Validation procedure

The validation procedure strictly followed the International standard ANSI/AAMI/ISO 81060-2:2013, and the same arm sequential method was adopted. For each subject, the validation team for each device consisted of two observers who performed the same number of measurements in this study. The observers used the reference sphygmomanometer to determine the subject’s BP. On each subject, three measurements were carried out with the BPM636 and three reference measurements were carried out by the observers. To stabilize the BP, each subject should rest for 10 min before taking measurements and wait at least 60 s between each measurement. The comparison of the value measured with the BPM636 and the BP values determined by observers were carried out consecutively on the same arm. It ensured that both measurements were carried out under identical conditions.

In the determination of the reference measurements, the observers determined the diastolic BP as the last audible Korotkoff sound (fifth phase or K5). Simultaneous auscultations were performed by two observers using the double stethoscope when measuring BP with the Korotkoff method. The two observers were blinded to each other’s readings and any pair of observers’ determinations with a difference greater than 4 mmHg shall be excluded.

All data from a subject shall be excluded if any two-reference SBP determinations differ more than 12 mmHg or if any two-reference DBP determinations differ more than 8 mmHg.

### Ethics

The test device BPM636, an arm type BP monitor for adult use designed and manufactured by AViTA Corporation, Ltd., had obtained a medical device license issued by Taiwan Food and Drug Administration. The central ethics committee B at the NTUH approved this study on 20 June 2018 (NTUH-REC NO.: 201805046RSB).

### Statistical analysis

The requirements of ANSI/AAMI/ISO Universal Standard (ISO 81060-2:2013) were strictly followed. Data analysis of systolic and diastolic BP shall meet the following two criteria, respectively. For criterion 1, for each paired determination, the difference between the test device and the reference readings (mean of two-reader-readings) was calculated. Across all reading determinations, the overall mean and SD of all differences shall not be greater than 5 and 8 mmHg, respectively. Furthermore, all differences were plotted against their respective means of paired determination using the Bland–Altman approach. For criterion 2, for each subject, the ‘averaged difference’ of paired determinations was calculated. Across all subjects, the SDs of all ‘averaged differences’ were further compared with the maximum permissible SD (which is a function of the overall mean) [18]. Microsoft Excel 2010 (Microsoft, Redmond, Washington, USA) was used for statistical analysis.

## Results

The subject’s statistics have two groups, all groups follow IS0 81060-2:2013 [18].

The AViTA BPM636 successfully passed criterion 1 and 2 in the ANSI/AAMI/ISO 81060-2 noninvasive sphygmomanometers validation protocol, section 5.2.4.1, the same arm sequential method.

### Group 1

**Fig. 1:**
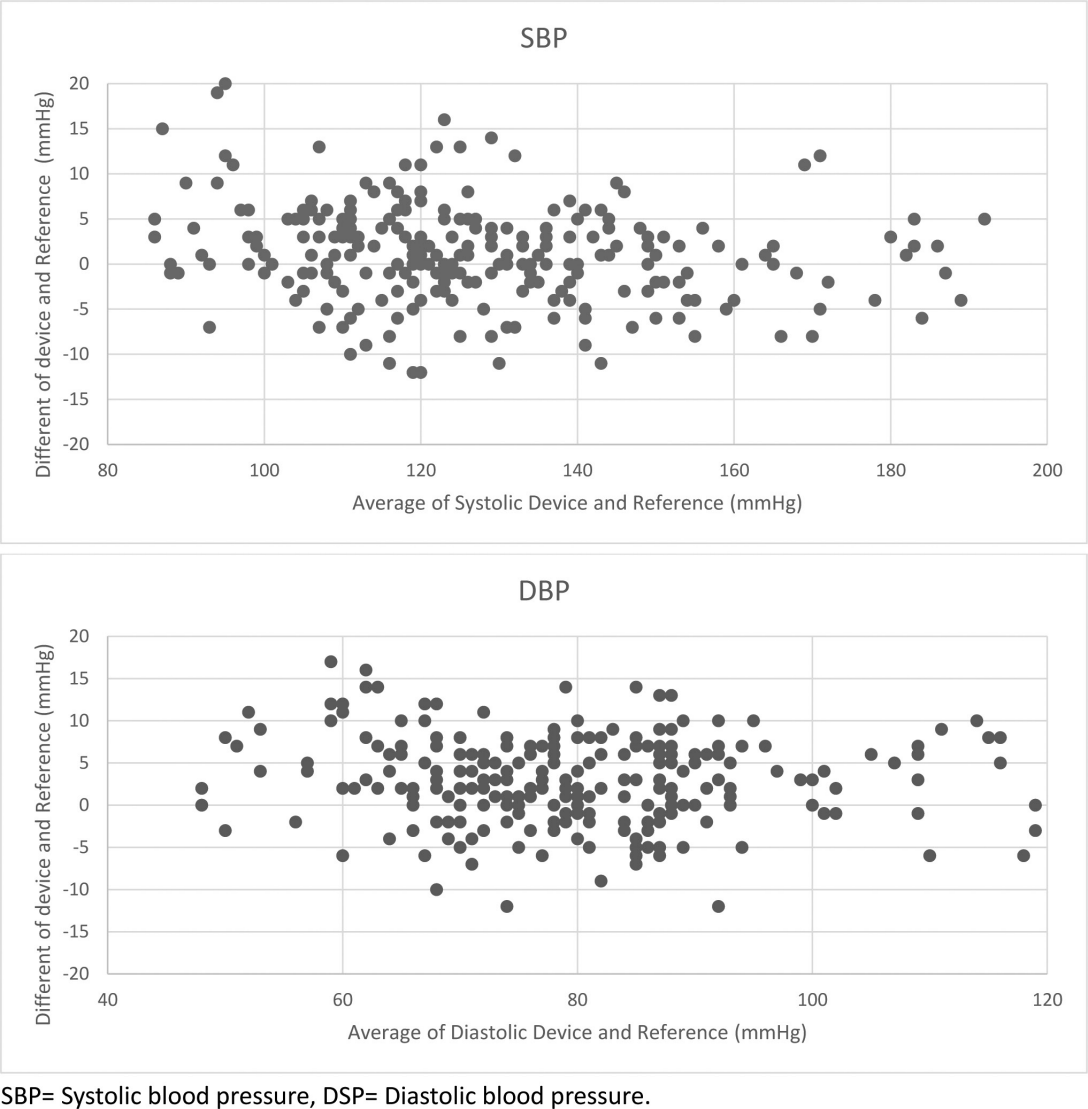
Standardized Bland–Altman scatter plots of the Test device and Reference BP differences against their mean value in adults.

The 85 adult subjects and 255 valid paired results systolic mean error of measurement was 1.1 mmHg and the SD was 5.49 mmHg; Diastolic mean error of measurement was 2.9 mmHg and the SD was 5.17 mmHg passed Criterion1 shown in Table 4. Standardized Bland–Altman scatter plots of the differences in test-reference BP values against their mean value in adult are shown in Fig. 1.

**Table 4:** Validation of the results according to protocol requirements (criterion 1)

Criterion 1	Mean error of measurement	SD	Result
Group 1 (adult, *N* = 85)			
Requirement	≦5 mmHg	≦8 mmHg	
SBP	1.1 mmHg	5.49 mmHg	Pass
DBP	2.9 mmHg	5.17 mmHg	Pass
Group 2 (pregnancy, *N* = 46)			
Requirement	≦5 mmHg	≦8 mmHg	
SBP	−2.2 mmHg	5.93 mmHg	Pass
DBP	1.5 mmHg	4.92 mmHg	Pass

The 85 adult subjects averaged paired result systolic mean error of measurement was 1.1 mmHg and the SD was 4.45 mmHg; diastolic mean error of measurement was 2.9 mmHg and the SD was 4.20 mmHg passed criterion 2 shown in Table 5.

**Table 5:** Validation the results according to protocol requirements (criterion 2)

Criterion 2	SBP	DBP	Result
Group 1 (adult, *N* = 85)			
Requirement	≦1.1 ± 6.89 mmHg	≦2.9 ± 6.30 mmHg	
Adult	1.1 ± 4.45 mmHg	2.9 ± 4.20 mmHg	Pass
Group 2 (pregnancy, *N* = 46)			
Requirement	≦1.1 ± 6.58 mmHg	≦1.5 ± 6.78 mmHg	
Pregnancy	−2.2 ± 4.66 mmHg	1.5 ± 3.96 mmHg	Pass

Mean error of measurement ± SD.

### Group 2

**Fig. 2:**
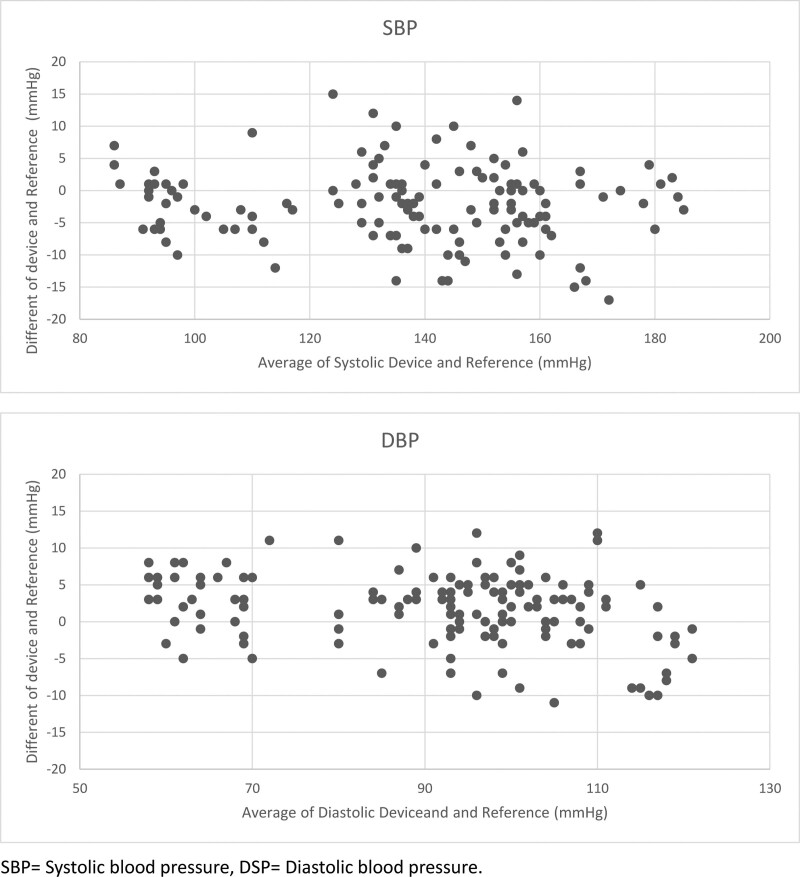
Standardized Bland–Altman scatter plots of the test device and reference BP differences against their mean value in pregnancy women.

The 46 pregnant subjects and 138 valid paired results systolic mean error of measurement of −2.2 mmHg and a SD of 5.93 mmHg. Diastolic mean error of measurement was 1.5 mmHg and the SD was 4.92 mmHg passed criterion 1 shown in Table 4. Standardized Bland–Altman scatter plots of the differences in test-reference BP values against their mean value in adult are shown in Fig. 2.

The 46 adult subjects averaged paired result systolic mean error of measurement was −2.2 mmHg and SD was 4.66 mmHg; diastolic mean error of measurement was 1.5 mmHg and SD was 3.96 mmHg passed criterion 2 shown in Table 5.

## Discussion

Although the selection of a BP measuring device may be influenced by many factors, a fundamental requirement should be fulfilled that it gives accurate measurements. Hypertension in pregnant women is common and has been reported to occur in near 10% of pregnancies [19], accurate BP measurements is the leading importance in diagnosing and monitoring high-risk pregnant women. Self-monitoring of BP is increasingly popular with patients, the general public and health professionals and pregnant patients [20]. Few research aimed in the validation of home BP device in specific pregnant population [21–26], even by using ANSI/AAMI/ISO 81060-2:2013 or later version [25,26]. Moreover, rare studies testing home BP devices among pregnant women with pre-eclampsia were performed without a protocol violation [16–18]. Some finding showed the BP monitor device may underestimate systolic BP measurements in patients with pre-eclampsia by using ANSI/AAMI/ISO 81060-2:2013 [16–18].

Although the updated version of the ISO 81060-2 has been released in November of 2018 [27], and this study was approved by the central ethics committee B at the NTUH on 20 June 2018, which was earlier than the updated version publication. This was the reason that this study followed the older version of ISO 81060-2. However, we did consider that ISO 81060-2:2018 has released during this study recruitment from 23 October 2018 to 25 September 2019, therefore the recruitment did refer to the ISO 81060-2:2018, and the results also demonstrated that the AViTA Arm Type BP Monitor passed the validation for both systolic and diastolic BPs according to the International Standard ISO 81060-2:2018, shown in Supplementary data, Supplemental digital content 1, http://links.lww.com/BPMJ/A194.

This study is to provide information on the accuracy of the AViTA BPM636 measurement in adult and pregnant populations. The validation study was performed according to the ANSI/AAMI/ISO 81060-2:2013 guidelines. Results showed that the AViTA BPM636 fulfilled validation criteria 1 and 2 in adult population and pregnant women.

### Conclusion

The results of this study demonstrated that the AViTA BPM636 passed the validation for both systolic and diastolic BPs according to the International Standard ANSI/AAMI/ISO 81060-2:2013 and this device can therefore be recommended for use not only in the adult and also in pregnant population.

## Acknowledgements

AViTA Corporation, Taiwan, provided funding for this study, the authors acknowledge the persons responsible for performing the actual measurements: Jessica Kang and Chin, K. C., C.N.L., and I.C.H. organized the study from a logistical perspective (patient recruitment, scheduling, alignment with AViTA and oversight of the measurements). C.N.L. was responsible for the ethics committee submission and approval. C.K.W. performed the statistics and I.C.H., wrote the initial draft of the article. All authors revised the article for important intellectual content and took responsibility for their own work. All agreed on the final version that was submitted.

This clinical validation was carried out at request by National Taiwan University Hospital (NTUH). Because there have professional observers and a quiet environment. That is to promote clinical validation accuracy.

### Conflicts of interest

I.C.H. is employed by and receives a salary from AViTA Corporation, the manufacturer of AViTA BPM636. For the remaining authors, there are no conflicts of interest.

## Supplementary Material


