# Supplementary material for: Validation of the AViTA BPM636 upper arm blood pressure monitor in adults and pregnant women according to the ANSI/AAMI/ISO 81060-2:2013
Source: Blood Press Monit. 2023 Apr 5;28(4):215–20. doi: 10.1097/MBP.0000000000000648 (PMC10309106; doi:10.1097/MBP.0000000000000648)
Supplement: Supplementary file 1 [file bpmj-28-215-s001.pdf]

Supplementary data

Conformity assessment with ISO 81060-2:2018 + A1:2020

1. Purpose

   The evaluation uses the clinical investigation data(NTUH-REC No.:201805046RSB) of ISO 81060-2:2013 non-invasive sphygmomanometers-Part2 Clinical investigation of intermittent automated measurement type, and evaluates its compliance with the specifications in accordance with the new version of the specification ISO 81060-2:2018+A1: 2020.
2. Scope

   The scope for AViTA arm sphygmomanometer with the same Intended for use, main components (such as cuff, sensor, microcontroller, etc.), and algorithm.
3. Requirements
   - NTUH-REC No.:201805046RSB Clinical investigation Raw data
   - ISO 81060-2:2013 Non-invasive sphygmomanometers-Part2 Clinical investigation automated measurement type
   - ISO 81060-2:2018 Non-invasive sphygmomanometers-Part2 Clinical investigation of intermittent automated measurement type
   - ISO 81060-2:2020 Non-invasive sphygmomanometers-Part2 Clinical investigation of intermittent automated measurement type AMENDMENT 1
4. Analysis and Results

Table A IS0 81060-2 Section

IS0 81060-2:2013 Section	IS0 81060-2:2018+A1:2020 Section	Clinical Data Results	Pass	Fail
**5.1.1 Number**<br>An auscultatory REFERENCE SPHYGMOMANOMETWE CLINICAL INVESTIGATION shall consist of a minimum of 85 subjects. If not otherwise specified, at least three valid paired BLOOD PRESSURE values shall be taken for each subject. There shall be a minimum of 255 valid paired BLOOD PRESSURE values.	**5.1.1 Number**<br>An auscultatory REFERENCE SPHYGMOMANOMETWE CLINICAL INVESTIGATION shall consist of a minimum of 85 subjects. If not otherwise specified, at least three valid paired BLOOD PRESSURE values shall be taken for each subject. There shall be a minimum of 255 valid paired BLOOD PRESSURE values.	85 Subjects , total 255 valid paired (Three valid paired for each subject)	V	
**5.1.2 Gender distribution**<br>At least 30 % of the subjects shall be male and At least 30 % of the subjects shall be female.	**5.1.2 Gender distribution**<br>At least 30 % of the subjects shall be male and At least 30 % of the subjects shall be female.	45 (53%) male and 40 (47%) female	V	
**5.1.3 Age distribution**	**5.1.3 Age distribution**	All subjects Age	V	

IS0 81060-2:2013 Section	IS0 81060-2:2018+A1:2020 Section	Clinical Data Results	Pass	Fail
For a sphygmomanometer intended for use on adults or adolescent patients, the age of every subject included in the clinical investigation shall be greater than 12 years.	For a sphygmomanometer intended for use on adults or adolescent patients, the age of every subject included in the clinical investigation shall be greater than 12 years.	greater than 12 years.		
**5.1.4 Limb size distribution**<br>For a sphygmomanometer intended for use with a single cuff size:<br>- at least 40 % of the subjects shall have a limb circumference which lies within the upper half of the specified range of use of the cuff and at least 40 % of the subjects shall have a limb circumference within the lower half of the specified range of use of the cuff	**5.1.4 Limb size distribution**<br>**Limb circumferences shall be distributed as follows:**<br>**- at least 20% of the subjects shall have a limb circumference which lies within each quarter of the TOTAL LIMB CIRCUMFERENCE RANGE.**	**- Subjects Arm size range between 22cm to 27cm is 24%.**<br>**- Subjects Arm size range between 27cm to 32cm is 35%.**<br>**- Subjects Arm size range between 32cm to 37cm is 21%**<br>**- Subjects Arm size range between 37cm to 42cm is 20%.**	V	
-at least 20 % of the subjects shall have a limb circumference which lies within the upper quarter of the specified range of use of the cuff and at least 20 % of the subjects shall have a limb circumference within the lower quarter of the specified range of use of the cuff.	**-at least 10% of the subjects shall have a limb circumference which lies within the highest octile of the TOTAL LIMB CIRCUMFERENCE RANGE.**<br>**-at least 10% of the subjects shall have a limb circumference which lies within the lowest octile of the TOTAL LIMB CIRCUMFERENCE RANGE.**	**- Subjects Arm size range highest than 39.5cm is 10%.**<br>**- Subjects Arm size range lowest than 24.5cm is 14%.**	V	
**5.1.5 Blood pressure distribution**<br>At least 5 % of the reference blood pressure readings shall have a systolic blood pressure ≤100 mmHg (13,33 kPa).	**5.1.5 Blood pressure distribution**<br>At least 5 % of the reference blood pressure readings shall have a systolic blood pressure ≤100 mmHg (13,33 kPa).	11% of the reference blood pressure readings have a systolic blood pressure ≤100 mmHg	V	

IS0 81060-2:2013 Section	IS0 81060-2:2018+A1:2020 Section	Clinical Data Results	Pass	Fail
At least 5 % of the reference blood pressure readings shall have a systolic blood pressure ≥160 mmHg (21,33 kPa).	At least 5 % of the reference blood pressure readings shall have a systolic blood pressure ≥160 mmHg (21,33 kPa).	9% of the reference blood pressure readings have a systolic blood pressure ≥160 mmHg	V	
At least 20 % of the reference blood pressure readings shall have a systolic blood pressure ≥140 mmHg (18,66 kPa).	At least 20 % of the reference blood pressure readings shall have a systolic blood pressure ≥140 mmHg (18,66 kPa).	25% of the reference blood pressure readings have a systolic blood pressure ≥140 mmHg	V	
At least 5 % of the reference blood pressure readings shall have a diastolic blood pressure ≤60 mmHg (8,0 kPa).	At least 5 % of the reference blood pressure readings shall have a diastolic blood pressure ≤60 mmHg (8,0 kPa).	8% of the reference blood pressure readings have a diastolic blood pressure ≤60 mmHg	V	
At least 5 % of the reference blood pressure readings shall have a diastolic blood pressure ≥100 mmHg (13,33 kPa).	At least 5 % of the reference blood pressure readings shall have a diastolic blood pressure ≥100 mmHg (13,33 kPa).	8% of the reference blood pressure readings have a diastolic blood pressure ≥100 mmHg	V	
At least 20 % of the reference blood pressure readings shall have a diastolic blood pressure ≥85 mmHg (11,33 kPa).	At least 20 % of the reference blood pressure readings shall have a diastolic blood pressure ≥85 mmHg (11,33 kPa).	35% of the reference blood pressure readings have a diastolic blood pressure ≥85 mmHg	V	
**5.1.6 Special patient populations**<br>Note: Clause 7 has a specific example of a special patient population with specific requirements.	**5.1.6 Special patient populations**<br>Note: Clause 7 has a specific example of a special patient population with specific requirements	Please refer to Clause 7.	V	
**7 Pregnant (including pre-eclamptic) PATIENT populations**<br>A sphygmomanometer that is intended for use in pregnant patients shall undergo clinical investigation in that patient population. If the sphygmomanometer has	**7 Pregnant (including pre-eclamptic) PATIENT populations**<br>A sphygmomanometer that is intended for use in pregnant patients shall undergo clinical investigation in that patient population. If the sphygmomanometer has	Group1 passed 5.1 and 5.2 and additional 46 pregnant patients (Group2)	V	

IS0 81060-2:2013 Section	IS0 81060-2:2018+A1:2020 Section	Clinical Data Results	Pass	Fail
passed the clinical investigation according to the requirements given in 5.1 and 5.2, then it shall undergo clinical investigation according to 5.2 in at least an additional 45 pregnant patients.	passed the clinical investigation according to the requirements given in 5.1 and 5.2, then it shall undergo clinical investigation according to 5.2 in at least an additional 45 pregnant patients.			
For any clinical investigation for pregnant patients, the patient population shall be equally distributed, ±1 patient, into the following three subgroups:<br>a) normotensive pregnant PATIENTS beyond the first trimester with SYSTOLIC BLOOD PRESSURE <140 mmHg (18,66 kPa) and DIASTOLIC BLOOD PRESSURE <90 mmHg (12,00 kPa);	For any clinical investigation for pregnant patients, the patient population shall be equally distributed, ±1 patient, into the following three subgroups:<br>a) normotensive pregnant PATIENTS beyond the first trimester with SYSTOLIC BLOOD PRESSURE <140 mmHg (18,66 kPa) and DIASTOLIC BLOOD PRESSURE <90 mmHg (12,00 kPa);	15 normotensive pregnant PATIENTS	V	
b) hypertensive pregnant PATIENTS without proteinuria > 300 mg in 24 h and with SYSTOLIC BLOOD PRESSURE ≥140 mmHg (18,66 kPa) or DIASTOLIC BLOOD PRESSURE ≥ 90 mmHg (12,00 kPa)	b) hypertensive pregnant PATIENTS without proteinuria > 300 mg in 24 h and with SYSTOLIC BLOOD PRESSURE ≥140 mmHg (18,66 kPa) or DIASTOLIC BLOOD PRESSURE ≥ 90 mmHg (12,00 kPa)	16 hypertensive pregnant PATIENTS	V	
c) pre-eclampsia, PATIENTS with proteinuria >300 mg in 24 h and DIASTOLIC BLOOD PRESSURE ≥ 90 mmHg (12,00 kPa).	c) pre-eclampsia, PATIENTS with proteinuria >300 mg in 24 h and with **SYSTOLIC BLOOD PRESSURE ≥140 mmHg (18,66 kPa)** or DIASTOLIC BLOOD PRESSURE ≥ 90 mmHg	15 pre-eclampsia PATIENTS	V	

IS0 81060-2:2013 Section	IS0 81060-2:2018+A1:2020 Section	Clinical Data Results	Pass	Fail
	(12,00 kPa).			
5.2.4.1.2 Data analysis<br>a) Criterion 1<br>ⅰ) According to blood pressure mean value of differences, with in or equal to ±5.0mmHg (±0.67kPa).<br>ⅱ)According to blood pressure standard deviation of differences, no greater than 8.0mmHg (1.07kPa).	5.2.4.1.2 Data analysis<br>a) Criterion 1<br>ⅰ) According to blood pressure mean value of differences, with in or equal to ±5.0mmHg (±0.67kPa).<br>ⅱ)According to blood pressure standard deviation of differences, no greater than 8.0mmHg (1.07kPa).	Systolic-<br>Mean: 1.10 mmHg<br>Standard deviation: 5.49mmHg<br>Diastolic:<br>Systolic-<br>Mean: 2.90 mmHg<br>Standard deviation: 5.17mmHg	V	
b) Criterion 2<br>1) For the SYSTOLIC BLOOD PPRESSURE and DIASTOLIC BLOOD PRESSURE for each of m subjects, the standard deviation Sm of the averaged paired DETERMINATIONS per subject of the SPHYGMOMANOMETER-UNDER-TEST and of the observers' readings with the REFERENCE SPHYGMOMANOMETER shall meet the criteria listed:<br>ⅰ) Table1; or<br>ⅱ) Table2	b) Criterion 2<br>1) For the SYSTOLIC BLOOD PPRESSURE and DIASTOLIC BLOOD PRESSURE for each of m subjects, the standard deviation Sm of the averaged paired DETERMINATIONS per subject of the SPHYGMOMANOMETER-UNDER-TEST and of the observers' readings with the REFERENCE SPHYGMOMANOMETER shall meet the criteria listed:<br>ⅰ) Table1; or<br>ⅱ) Table2	Systolic-<br>Mean: 1.10 mmHg<br>Standard deviation: 4.45mmHg (<6.89mmHg)<br>Diastolic:<br>Systolic-<br>Mean: 2.90 mmHg<br>Standard deviation: 4.20mmHg (<6.30mmHg)	V	

Table 1 — Averaged subject data acceptance (criterion 2) in mmHg

$\bar{x}_n$	Maximum permissible standard deviation, s_m, as function of, $\bar{x}_n$ mmHg									
	**0,0**	**0,1**	**0,2**	**0,3**	**0,4**	**0,5**	**0,6**	**0,7**	**0,8**	**0,9**
**±0,**	6,95	6,95	6,95	6,95	6,93	6,92	6,91	6,90	6,89	6,88
**±1,**	6,87	6,86	6,84	6,82	6,80	6,78	6,76	6,73	6,71	6,68
**±2,**	6,65	6,62	6,58	6,55	6,51	6,47	6,43	6,39	6,34	6,30
**±3,**	6,25	6,20	6,14	6,09	6,03	5,97	5,89	5,83	5,77	5,70
**±4,**	5,64	5,56	5,49	5,41	5,33	5,25	5,16	5,08	5,01	4,90
**±5,**	4,79	—	—	—	—	—	—	—	—	—
EXAMPLE For mean of ±4,2 mmHg, the maximum permissible standard deviation is 5,49 mmHg.										

Table 2 — Averaged subject data acceptance (criterion 2) in kPa

$\bar{x}_n$	Maximum permissible standard deviation, s_m, as function of, $\bar{x}_n$ kPa									
	**0,000**	**0,010**	**0,020**	**0,030**	**0,040**	**0,050**	**0,060**	**0,070**	**0,080**	**0,090**
**±0,0**	0,926 6	0,926 6	0,926 6	0,926 6	0,926 6	0,924 6	0,923 3	0,922 3	0,921 3	0,920 3
**±0,1**	0,919 3	0,918 3	0,917 3	0,916 3	0,915 2	0,913 8	0,911 9	0,909 9	0,907 9	0,905 9
**±0,2**	0,903 9	0,900 7	0,898 9	0,897 0	0,894 6	0,890 6	0,887 8	0,885 5	0,882 6	0,878 5
**±0,3**	0,875 6	0,872 3	0,867 9	0,864 1	0,860 1	0,856 2	0,851 9	0,847 1	0,841 4	0,837 4
**±0,4**	0,833 3	0,828 3	0,822 6	0,816 9	0,811 9	0,805 9	0,799 9	0,793 3	0,785 3	0,779 3
**±0,5**	0,773 9	0,766 9	0,759 9	0,753 1	0,746 3	0,738 8	0,731 9	0,723 7	0,715 7	0,707 7
**±0,6**	0,699 9	0,689 1	0,680 2	0,672 3	0,667 0	0,659 5	0,648 8	0,638 6	—	—
EXAMPLE For mean of ±0,520 kPa, the maximum permissible standard deviation is 0,759 9 kPa.										

5. Conclusion

The results of the present study demonstrated that the AViTA Arm Type Blood Pressure Monitor passed the validation for both systolic and diastolic blood pressures according to the International Standard ISO 81060-2:2018+A1:2020.
